# Impact of a dTcaP booster vaccine awareness campaign initiated by the French national health insurance for adults aged 25 years in 2021

**DOI:** 10.1186/s12913-023-09805-w

**Published:** 2023-08-23

**Authors:** Aline Hurtaud, Capucine Coomans, Brigitte Vuillemin, Akima Benamar, Maxime Couraud, Bach-Nga Pham, Stéphane Sanchez, Coralie Barbe

**Affiliations:** 1 Department of General Medicine, University of Reims Champagne-Ardenne UFR Medicine, 51 rue Cognacq Jay, Reims, 51100 France; 2 Caisse Primaire d’Assurance Maladie des Ardennes, 14 Avenue Georges Corneau, Charleville- Mézières, 08100 France; 3 Research on Health University Department, University of Reims Champagne-Ardenne UFR Medicine, 51 rue Cognacq Jay, Reims, 51100 France

**Keywords:** Vaccination coverage, Diphtheria-tetanus-pertussis-polio, Patient reminders, Vaccination, Health insurance, Booster vaccine

## Abstract

**Background:**

Vaccination schedules differ from country to country. In France, the diphtheria, tetanus, pertussis, poliomyelitis (dTcaP) booster vaccine coverage for adults aged 25 has been lower than those recommended. We evaluated the impact of an awareness campaign undertaken by the French national health insurance system in 2021.

**Methods:**

A randomized, controlled study with adults residing in the Ardennes region was conducted to evaluate the effect on vaccine coverage of the booster vaccine reminder campaign carried out via letter and/or email and/or SMS. The randomization unit was the municipal administrative area (canton). Ten cantons were grouped into the intervention group (INT) and nine were the control group (CON). Outcomes were the booster vaccine delivery and the consultation of a general practitioner (GP) within 12 months (since the French national health insurance running the campaign suggested patients to consult their GP).

**Results:**

A total of 1,975 adults were included (INT: 67.3% vs. CON: 32.7%). Of them, 331 received a booster vaccine (INT: 17.4% vs. CON: 15.5%; p = 0.29), and 1,442 consulted a GP (INT: 73.7% vs. CON: 76.8%; p = 0.14). Those who consulted a GP had more frequent vaccine delivery (INT: 19.1% vs. CON: 10.5%; p < 0.0001).

**Conclusions:**

This study found that the awareness campaign run by the French national health insurance did not improve the uptake of the dTcaP booster and that there was a low rate of vaccinated adults aged 25 years. A GP consultation was associated with dTcaP booster vaccine delivery which may show that there is a need of involving GPs in vaccination follow-ups. Patients recognize GPs as providers of credible information and they may play a key role in individualized preventive healthcare actions. Systematic consultations with GPs for follow-up could be proposed to insured adults aged 25 years in the future.

## Background

Vaccination schedules differ from country to country and are followed according to local recommendations. According to literature, the best scheduling model in Europe for the diphtheria, tetanus, pertussis and poliomyelitis (DTPP) vaccination in children as well as the influenza vaccination for people over 65 years is still unestablished [1, 2]. In France, the Ministry of Health annually updates immunization schedules after consulting with public health authorities such as the *Haute Autorité de Santé* (HAS), then sets the applicable immunization recommendations according to age [3].

The initial vaccination in the DTPP schedule is mandatory with two injections of the diphtheria, tetanus, pertussis, poliomyelitis vaccine for children (DTCaP) at two and four months of age followed by boosters at 11 months and six years. A booster dose of the vaccine for adults (dTcaP) is then recommended for people aged 12, 25, 45 and 65 years old followed by a dose every 10 years thereafter. This scheduling is displayed in the health notebook that is systematically distributed to the parents of every child born in France. To date, long-term vaccination proposal campaigns do not exist, and vaccination uptake is based on the medical relationship between practitioners and patients. Moreover, vaccines must be prescribed by a medical practitioner and are reimbursed at 65% by the health insurance.

The public health policy in France sets to achieve at least 95% vaccination recommendation coverage (all vaccines except for influenza) at the appropriate ages [4]. To date, limited data are available on adult vaccination coverage and existing literature states that vaccination coverage is poor among adults. In the United States, a national, cross-sectional household survey of the non-institutionalized civilian population showed inadequate immunization coverage for all vaccines with 64.5% of 19- to 49-year-olds reportedly having received a tetanus toxoid-containing booster vaccine within the last 10 years [5]. In France, coverage of the dTcaP booster vaccine for patients over sixteen years of age was estimated at 50.5% in 2012 [6–8]. In 2017, Marchal et al. evaluated the dTcaP booster vaccine coverage at 46.6% in adults aged 29 years based on data from the French national health insurance system known as the *Caisse Primaire d’Assurance Maladie* (CPAM) [9]. Similarly, in a study of French general practitioners (GPs), 40% of adults (with a mean age of 44 ± 15 years) were estimated not to be up to date with this booster, citing that a lack of coverage was mostly due to forgetfulness (24.2%) and negligence (15.2%) [10].

The effectiveness of awareness campaigns for adults is often questionable and presents a public health concern. In a systematic review, vaccination reminders and follow-up interventions via telephone call, email, letter or SMS for people in different age groups and countries found increased vaccination coverage by an average of 8% [11]. In regard to vaccination program interventions, another study showed an improvement in adolescent vaccination coverage when parents received email reminders for four recommended vaccinations (meningococcal, dTcaP, HPV and varicella). Furthermore, schools that reminded students about influenza vaccinations by email and/or letter had better vaccination coverage compared with email reminders alone. Thus, the combination of the two types of reminders were reported to be more effective [12]. The the objective of this study was to evaluate the impact of an awareness campaign initiated by the CPAM for the dTcaP booster vaccine for adults aged 25 years in France.

## Methods

### Study design

A prospective, randomized, controlled study was conducted in the Ardennes region in 2021. Participants included were those aged 25 years in 2020, residing in the region in June 2021 and who had not had a dTcaP booster vaccine dispensed from a pharmacy between January 1, 2019 and June 1, 2021. Those that were not affiliated with the general French health insurance system were excluded.

### Study variables

The randomization unit was a municipal administrative area known as the canton. Among the 19 cantons in the Ardennes region, participants were pooled out of 10 cantons that were randomly selected to constitute the intervention group (INT) and of nine cantons that made up the control group (CON). The choice of the canton as the randomization unit made it possible to limit contamination bias.

**Fig. 1:**
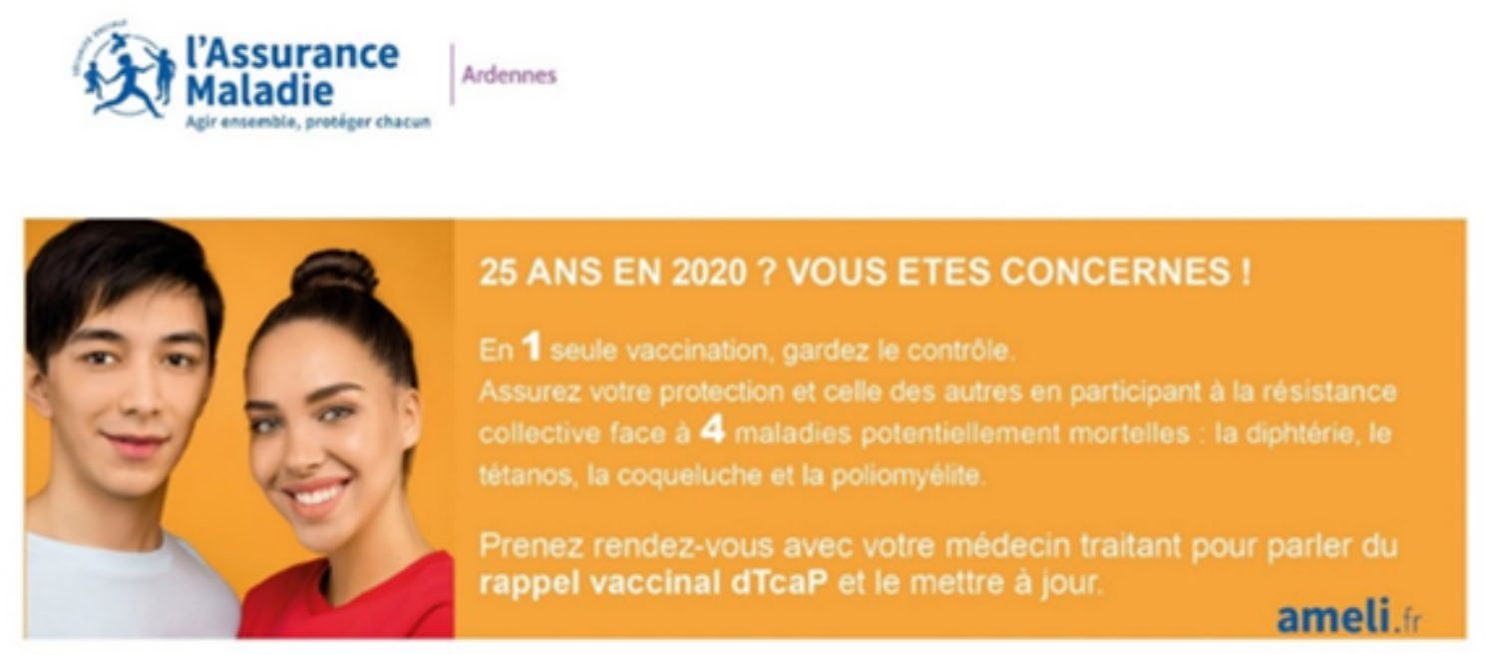
Information of the awareness campaign for the dTcaP booster vaccine disseminated by the French national health insurance to French adults aged 25 years (intervention group) residing in the Ardennes region on June 1, 2021

Participants (aged 25 in 2020) in the INT group received information regarding the dTcaP booster vaccine (Fig. 1) by the local CPAM Pole via letter and/or email and/or SMS according to their given consent of which type of method they agreed to be contacted by. The information was produced in accordance with the CPAM charter and was developed by a multidisciplinary team comprised of GPs and public health doctors. The messages sent to participants began by identifying the target audience with: “25 years old in 2020? you’re concerned”, then, a reminder with the following text: “With just 1 vaccination, stay in control. Protect yourself and others by contributing to collective resistance to four potentially fatal diseases: diphtheria, tetanus, pertussis and poliomyelitis”. Lastly, the target audience were advised to consult their GP with the following message: “Make an appointment with your GP to discuss your dTcaP booster vaccination and bring it up to date”. The awareness campaign was disseminated all at once on June 1, 2021 for the INT group. In June 2022, those in the CON group received the same information via letter and/or email, and/or SMS according to their given consent of which type of method they agreed to be contacted by and after granting retroactive consent to use their data and participation in the study. The booster vaccine was not issued in this study.

### Data collection

Data was collected on the dispensing of the dTcaP booster vaccine by pharmacies and pro-pharmacies within 12 months that the information by the CPAM had been disseminated. Outcomes included the dTcaP booster vaccine delivery (which was assessed using the Club Inter Pharmaceutic (CIP) codes corresponding to the vaccine in the CPAM database) and a consultation with a GP at least once within 12 months after the start of the awareness campaign for those who had already received the dTcaP booster vaccine. A GP consultation was an endpoint because the French national health insurance running the awareness campaign suggested that patients consult with their GP.

### Ethical considerations

Data processing was carried out in compliance with the French regulations, in particular the General Data Protection Regulation (GDPR) 2016/679 of the European Parliament and the Council of April 27, 2016 applicable since May 25, 2018 as well as the Data Protection Act of January 6, 1978 (amended in 2018). Participants’ non-opposition to the use of their data was collected. The study was registered in the public directory of the Health Data Hub (No. F20210521141129) and was approved by the Ethics Committee of the *Collège National des Généralistes Enseignants* (No.010721290 dated July 28, 2021).

### Statistical analysis

Data were described using numbers and percentages (%). The analysis of the outcomes consisted of a comparison between the percentage of dTcaP booster vaccine delivery and the percentage of GP visits in both the INT and CON groups using Chi-square tests. The significance level was set at 0.05. Statistical analysis was performed using SAS software (Version 9.4, SAS Inc., Cary, NC, USA).

## Results

**Fig. 2:**
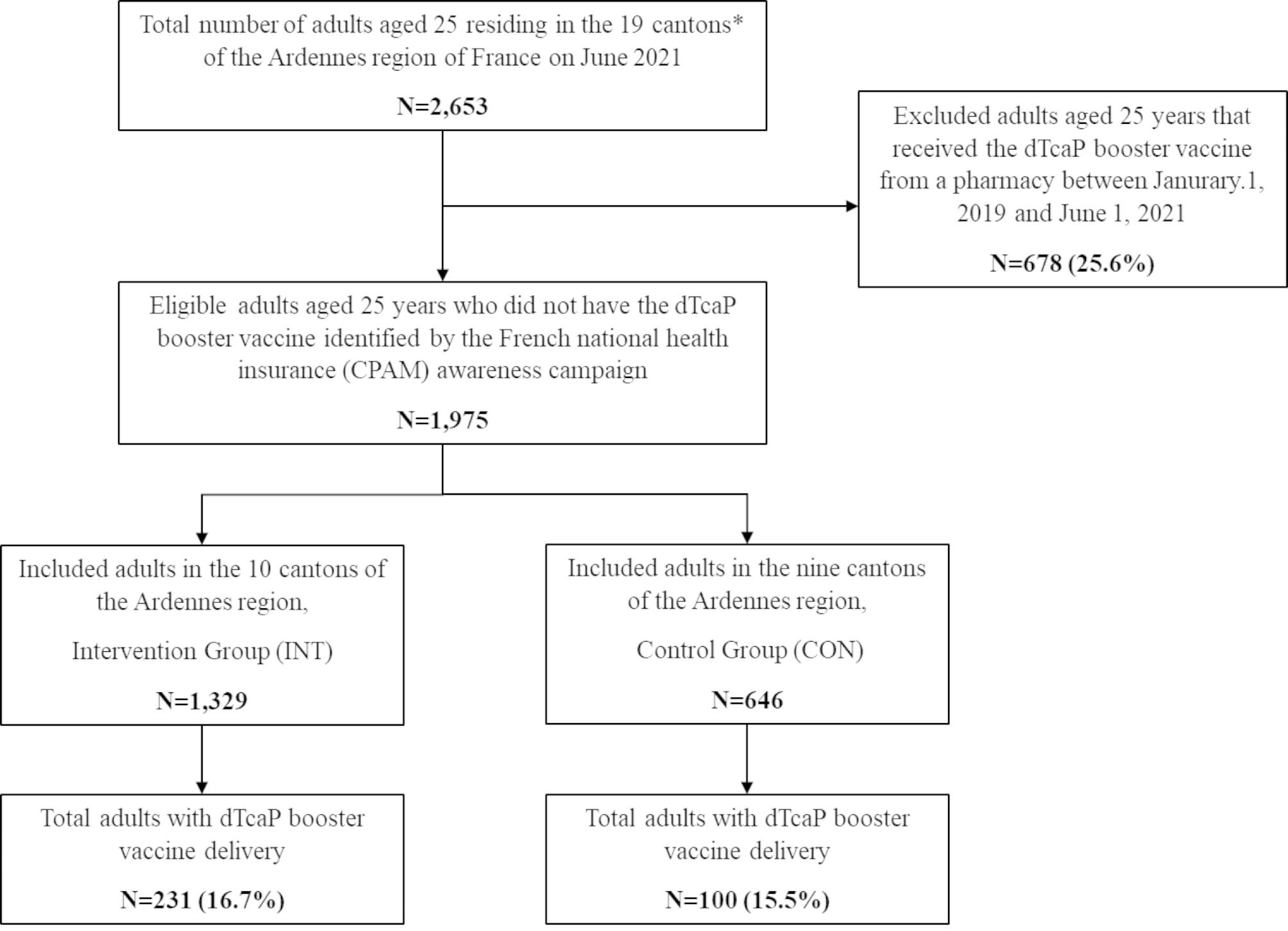
Study flow chart. *municipal administrative areas, CPAM; *Caisse Primaire d’Assurance Maladie*

Among the 2,653 adults included in this study, a total of 678 (25.6%) received a dTcaP booster vaccine from a pharmacy or pro-pharmacy in 2021. The remaining 1,975 eligible adults were identified by the local CPAM Pole (INT: 67.3% vs. CON: 32.7%) (Fig. 2).

The dTcaP booster vaccine delivery between June 2021 and June 2022 occurred for 331 (16.7%) adults and no significant difference concerning the rate of vaccination was found between the two groups (INT: 17.4% vs. CON: 15.5%; p = 0.29) (Table 1). Regarding the rate of GP consultations, 1,442 (73.0%) adults consulted a GP during the study period and no significant difference was found between the two groups (INT: 73.7% vs. CON: 76.8%; p = 0.14). Our study found that adults aged 25 years who had consulted a GP had a significantly more frequent delivery of the dTcaP booster vaccine (INT: 19.1% vs. CON: 10.5%; p < 0.0001).

**Table 1:** Outcomes of an awareness campaign initiated by the French national health insurance for the dTcaP booster vaccine for adults aged 25 years in the 19 cantons of the Ardennes region

	Delivery of the dTcaP booster vaccine N (%)	Non-delivery of the dTcaP booster vaccine N (%)	p-value
CPAM awareness campaign	331	1,644	0.29
Intervention group*	231 (17.4)	1,098 (82.6)	
*Control group*	100 (15.5)	546 (84.5)	
Consultation with a general practitioner†			< 0.0001
*Intervention group*	275 (19.1)	1,167 (80.9)	
*Control group*	56 (10.5)	477 (89.5)	

*Ten cantons were randomly selected to constitute the intervention group (INT) and nine cantons made up the control group (CON). †at least once within 12 months after the start of the awareness campaign. CPAM; *Caisse Primaire d’Assurance Maladie*

## Discussion

This study found that the awareness campaign run by the CPAM did not improve the uptake of the dTcaP booster for adults aged 25 years. These findings may be inconsistent with existing literature on the effectiveness of patient reminders for vaccinations [11] and in other areas of prevention. For cancer screening programs, several studies reported increased adherence through the use of email invitations including a meta-analysis study evaluating different strategies to increase the participation of women in breast cancer screening via letter, telephone calls, home visits and informational brochures [13].

Since this study was conducted in 2021, the effects of the COVID-19 pandemic may have limited the uptake of the dTcaP booster vaccine and corresponds with Rachlin et al., that found a 3% decrease for routine practices of other vaccinations in 2021 [14]. In other words, although the COVID-19 pandemic led to the succession of new vaccinations and boosters, the vaccination program to protect against COVID-19 and the associated lockdown restrictions may have competed with non-COVID-19 related booster vaccine delivery. In the Ardennes region, COVID-19 vaccination was well respected by the population with 93.5% of 25 to 29-year-olds being fully vaccinated [15]. Conversely, vaccination against COVID-19 in people over 18 years old on May 12, 2021 may have provided an opportunity for 25-year-olds to receive the dTcaP booster, therefore, increasing dTcaP vaccination coverage before the study period. Among those aged 25 years in 2020 residing in the region in June 2021, 25.6% received a dTcaP booster vaccine in the previous year. However, the number of those vaccinated during the study period was lower (16.7%).

As a health insurer, the CPAM is not entirely recognized among the French population for its preventive approaches, therefore, the awareness campaign may not have corresponded to the expectations of the participants. Some participants may not have paid sufficient attention to the information disseminated in the awareness campaign. In 2020, Plichon et al. studied the impact of the framing of a message and the arguments presented to explain changes in attitude towards vaccinations, intention to be vaccinated or to recommend a vaccination to relatives [16]. Their study was carried out with young adults aged 18 to 25 years and showed that the formulation of a message from a punitive point-of-view (non-vaccination is illegal) did not increase the intention of participants wanting to be vaccinated, whereas a positive message formulation (being vaccinated respects the law) led to a feeling of being protected by the law and a positive change was reported in the attitude towards vaccination as well as stronger intention for participants to be vaccinated. The wording of the information provided in the CPAM campaign was phrased in a gain-altruistic way and highlighted that being vaccinated protects one’s health and that of others. We surmised that a shorter or legally oriented formulation may have had a greater impact. It is also possible that participants in this study did not feel concerned by the generic and impersonal message provided.

The rate of vaccinated adults during the study period was low (16.7%). Over the last few years, vaccine hesitancy associated with negative feelings about the safety of vaccines has developed in France. According to Larson et al., in 2016 among 67 countries, 45.2% of the French population were skeptical about vaccine safety [17]. However, in the Vaccine Confidence Project, the latest analysis dating from 2020 and carried out in 27 European Union countries and the UK showed that slightly over half of the French population surveyed (51%) had overall confidence in vaccines (COVID-19 vaccinations excluded) [18].

Consultations with a GP was significantly associated with the uptake of the dTcaP booster vaccine. Patients recognize GPs as providers of credible information and they play a key role in individualized preventive healthcare actions, in which their influence on the acceptance of vaccines in children has already been shown [19, 20]. In 2004, Beytout et al. discussed the possibility of creating a specific GP appointment at fixed ages in order to allow both GPs and the general population to be up to date with vaccinations in France [10]. This was taken up by the French Minister of Health on September 19, 2022 in a bill covering the financing of national health insurance programs. Moreover, Goodwin et al. showed an increase in participation in colorectal cancer screening when an invitation letter was signed by a GP [21]. Therefore, one way of improving dTcaP vaccination coverage for 25-year-olds who participated in our study could be for the CPAM and GPs to work together. Multiple studies such as Blank et al. (2008) show that a recommendation from a healthcare provider is the most important driver to increase vaccination uptake [22]. Moreover, a survey in Ireland conducted by Giese et al., in 2013, identified that influenza vaccination was associated with a GP vaccination recommendation [23].

Regarding the strengths and limitations of this study, the originality of testing the impact of an awareness campaign on a large population via a randomized methodology posed a strength. However, we did not directly question the participants to identify the reasons why participants did not have the booster vaccine in the follow-up. For the INT group, we were unable to ascertain if information from the CPAM campaign was actually received and read, nor to assess their satisfaction with its content. The absence of an individual data collection on participants’ follow-up and healthcare pathways also did not allow us to take into consideration any possible confounding factors such as the professional setting (where some may have received a vaccination through an occupational medicine specialist), or to identify a routine follow-up provided by the GP.

## Conclusions

Vaccination coverage of the dTcaP booster vaccine for 25-year-olds was low in the Ardennes region in France in 2021 and the awareness campaign run by the CPAM did not improve vaccine uptake. A GP consultation was associated with dTcaP booster vaccine delivery which may show that there is a need of involving GPs in vaccination follow-ups. Systematic consultations with GPs for follow-ups could be proposed to insured adults aged 25 years in the future.

## Data Availability

The datasets used and/or analysed during the current study are available from the corresponding author on reasonable request.

## List of abbreviations

CIP: Club Inter Pharmaceutic
CON: control group
CPAM: *Caisse Primaire d’Assurance Maladie*
DTPP: diphtheria, tetanus, pertussis and poliomyelitis
DTCaP: diphtheria, tetanus, pertussis, poliomyelitis vaccine for children
dTcaP: diphtheria, tetanus, pertussis, poliomyelitis vaccine for adults
GP: general practitioner
HAS: *Haute Autorité de Santé*
INT: intervention group
